# Anthelminthic activity of glibenclamide on secondary cystic echinococcosis in mice

**DOI:** 10.1371/journal.pntd.0006111

**Published:** 2017-11-30

**Authors:** Julia A. Loos, María Sandra Churio, Andrea C. Cumino

**Affiliations:** 1 Laboratorio de Zoonosis Parasitarias, Departamento de Biología, Facultad de Ciencias Exactas y Naturales, Universidad Nacional de Mar del Plata (UNMdP), Mar del Plata, Argentina; 2 Consejo Nacional de Investigaciones Científicas y Técnicas (CONICET), Argentina; 3 Departamento de Química, Facultad de Ciencias Exactas y Naturales, Universidad Nacional de Mar del Plata (UNMdP), Mar del Plata, Argentina; 4 IFIMAR, Instituto de Investigaciones Físicas de Mar del Plata (CONICET-UNMdP), Argentina; University of Pennsylvania, UNITED STATES

## Abstract

Cystic echinococcosis (CE) is a worldwide parasitic zoonosis caused by the larval stage of *Echinococcus granulosus*. Current chemotherapy against this disease is based on the administration of benzimidazoles (BZMs). However, BZM treatment has a low cure rate and causes several side effects. Therefore, new treatment options are needed. The antidiabetic drug glibenclamide (Glb) is a second-generation sulfonylurea receptor inhibitor that has been shown to be active against protozoan parasites. Hence, we assessed the *in vitro* and *in vivo* pharmacological effects of Glb against the larval stage of *E*. *granulosus*. The *in vitro* activity was concentration dependent on both protoscoleces and metacestodes. Moreover, Glb combined with the minimum effective concentration of albendazole sulfoxide (ABZSO) was demonstrated to have a greater effect on metacestodes in comparison with each drug alone. Likewise, there was a reduction in the cyst weight after oral administration of Glb to infected mice (5 mg/kg of body weight administered daily for a period of 8 weeks). However, in contrast to *in vitro* assays, no differences in effectiveness were found between Glb + albendazole (ABZ) combined treatment and Glb monotherapy. Our results also revealed mitochondrial membrane depolarization and an increase in intracellular Ca^2+^ levels in Glb-treated protoscoleces. In addition, the intracystic drug accumulation and our bioinformatic analysis using the available *E*. *granulosus* genome suggest the presence of genes encoding sulfonylurea transporters in the parasite. Our data clearly demonstrated an anti-echinococcal effect of Glb on *E*. *granulosus* larval stage. Further studies are needed in order to thoroughly investigate the mechanism involved in the therapeutic response of the parasite to this sulfonylurea.

## Author summary

In this work we demonstrated the *in vitro* and *in vivo* efficacy of Glb against the larval stage of *Echinococcus granulosus*. At the cellular level, the drug triggered mitochondrial membrane depolarization and increased intracellular Ca^2+^ levels, thus affecting ATP generation in the parasite. In addition, since intracystic Glb concentrations were higher than those used in the external medium, we proposed that the drug might enter the cyst through cell surface transporters. The observed effect of the drug on the growth of hydatid cysts in mice leads to the consideration of a novel role of Glb in CE treatment. Therefore, our further studies will focus on the evaluation of ABZ formulations with enhanced bioavailability to achieve an improved *in vivo* anti-echinococcal effect using both drugs simultaneously.

## Introduction

Cystic echinococcosis (CE) is among the most serious and life-threatening helminth infections in humans worldwide [1]. This disease is caused by the larval stage of the dog-tapeworm *Echinococcus granulosus*. Not only it does affect approximately 2–3 million people around the world but it causes substantial economic losses to the livestock industry [2]. Currently, CE chemotherapy involves the use of benzimidazoles (BZMs), with albendazole (ABZ) the most commonly used. However, this treatment option is not curative and it often leads to side effects [3]. Therefore, further research should focus on the development of alternative therapies for CE. This includes the use of combination treatments; firstly, to increase therapeutic effectiveness, and secondly, to delay the emergence of possible resistance [4].

Glibenclamide (Glb) is a diarylsulfonylurea that has been widely used in the clinic to treat type 2 diabetes mellitus [5]. Furthermore, a great number of other pharmacological properties have been reported to be associated to this drug, such as anti-cancer [6], anti-proliferative [7, 8] and anti-inflammatory [9, 10] activities. This drug has a low cost (less than USD 0.10 per dose), it is non-toxic at doses commonly employed and it is readily available [11]. Glibenclamide has a high oral absorption but a low dissolution in gastric fluids and its pharmacokinetics depends on several transporters belonging to the solute carrier (SLC) and ABC superfamilies. Its uptake is mediated by the organic anion-transporting polypeptide (OATP, SLCO) family (preferentially by OATP2B1, OATP1B1 and OATP1B3). Its metabolism is through the cytochrome-P450 (CYP)-mediated oxidative pathways in the liver and its distribution and subsequent elimination is mediated by ABC transporters [12].

Glibenclamide exerts its mechanism of action by inhibiting ABC proteins with dissimilar functions, such as the sulfonylurea receptor–SURx, ABCC8–[13], which is associated with the pore-forming inwardly rectifying K^+^ channel–Kir6.X– (which together form the K_ATP_ channels), the cystic fibrosis transmembrane conductance regulator [14], the ABC1 transporter of immune cells [15], the P-glycoprotein (P-gp) [16] and the multidrug resistance-associated protein (MRP) (ABCC1) of cancer cells [17, 18]. Moreover, Glb causes modulation of mitochondrial permeability by action on different ion channels [19–21].

In the present study, we assessed the *in vitro* and *in vivo* effects of Glb on the viability and growth of *E*. *granulosus* larval stage. Our data clearly demonstrated that the drug possesses an *in vitro* anti-echinococcal activity against both protoscoleces and metacestodes. In addition, the observed effect of the drug on the growth of hydatid cysts in mice leads to the consideration of a novel role of Glb in CE treatment.

## Materials and methods

### Chemicals

Glibenclamide (INN) was obtained from Sigma-Aldrich (USA), JC-1 from Thermo Fisher Scientific (USA) and ABZ and albendazole sulfoxide (ABZSO) were kindly provided by Dr. C. Salomon (National University of Rosario, Argentina). For *in vitro* assays, Glb and ABZSO were kept as a 100 mM and a 100 μM stock solution in dimethyl sulfoxide (DMSO), respectively, and added to the medium either separately or in combination. For *in vivo* experiments, oil solutions of Glb and ABZ (corn oil, Sigma-Aldrich) were prepared every 2 days from solid drug and maintained under refrigeration (3–5°C).

### Ethics statement

Mice and bovine viscera were handled according to guidelines, management protocols and under the consent of the National Health Service and Food Quality (SENASA, Argentina), and in accordance with the 2011 revised form of The Guide for the Care and Use of Laboratory Animals published by the U.S. National Institutes of Health. The experimental protocols using parasite samples obtained from bovine viscera and infected mice with *E*. *granulosus* were evaluated and approved by the Animal Experimental Committee at the Faculty of Exact and Natural Sciences, Mar del Plata University (permit number: 2555-08-15).

### *In vitro* drug testing assays on larval stage of *E*. *granulosus*

Protoscoleces were removed aseptically from hydatid cysts of infected cattle slaughtered in the Liminal abattoir (official number: 3879) located in the Southeast of Buenos Aires, Argentina, as part of the normal work of the abattoir. Viable and morphologically intact protoscoleces (*n* = 3,000) were cultured using medium 199 (Gibco) supplemented with glucose (4 mg/ml) and antibiotics (penicillin, streptomycin and gentamicin 100 μg/ml) in 24-well culture plates under normal atmospheric conditions as we described in detail previously [22]. Murine cysts (with diameters ranging between 3 and 10 mm) were obtained from the peritoneal cavities of CF-1 mice 5 months after intraperitoneal infection with protoscoleces. Then, from 10 to 20 *E*. *granulosus* murine cysts per replica were incubated in Leighton tubes under the same culture conditions as described for protoscoleces [23]. *In vitro* protoscolex treatments were performed with 0.2, 2 and 10 mM Glb for 20 days while *in vitro* metacestode treatments were performed with 10, 50, 100 and 200 μM Glb, 2.5 μM ABZSO (equivalent to 0.84 μg/ml), and the combination of 10, 50, 100 and 200 μM Glb plus 2.5 μM ABZSO for 7 days [23]. Parasites incubated in culture medium containing DMSO were used as controls. *In vitro* protoscolex cultures were kept at 37°C with medium changes every 4 days. The protoscolex viability was determined every two days by the methylene blue exclusion test (at least 100 protoscoleces per replica were counted each time). The metacestode viability was assessed daily by trypan blue staining of detached germinal layers. Each experiment was performed in triplicate and repeated three times. All of the experiments were carried out until the viability of the control was lower than 90% or all treated parasites were dead.

### Assessment of mitochondrial membrane potential (ΔΨm)

Control and Glb-treated protoscoleces (200 μM Glb for 24 h) were incubated with 10 mg/ml JC-1 dye for 30 min at room temperature. After incubation, parasites were washed with 20 mM HEPES buffer, pH 7.2, and images were taken using a confocal microscope (Nikon Eclipse C1 Plus). The intensities of green (excitation/emission wavelength = 485/538 nm) and red (excitation/emission wavelength = 485/590 nm) fluorescence were analyzed for 20 individual protoscoleces from control and treated-samples. Images were analyzed using Image J software (NIH). The ratio of red to green fluorescence of JC-1 images was calculated using NIH Image J software (http://rsb.info.nih.gov/ij/).

### Detection of intracellular calcium levels in *E*. *granulosus* protoscoleces

Changes in intracellular-free Ca^2+^ concentration ([Ca^2+^]_i_) were fluorometrically monitored using Fluo3 acetoxymethylester (Fluo3-AM) probe [24]. Experiments were carried out with 5 x 10^3^ protoscoleces incubated under control conditions or treated with 200 μM Glb for 2 h. Then, the parasites were incubated with a solution containing 10 μM Fluo3-AM, 1 mM CaCl_2_, 0.1% v/v pluronic F-127 and 2 mM probenecid (Sigma, USA) for 30 min at 4° C in the dark. Subsequently, the protoscoleces were washed thrice in 20 mM HEPES buffer, pH 7.2 (without calcium) and the fluorescence was immediately registered with a spectrofluorimeter (model F-4500; Hitachi) every minute for 2 h. Excitation was provided by the 488 nm line of a krypton-argon laser and the emitted fluorescence was collected using band pass filters: 505–530 nm. Each experiment was individually corrected for autofluorescence. Protoscoleces were also subsequently imaged with an inverted confocal laser scanning microscope (Nikon, Confocal Microscope C1).

### Determination of glibenclamide levels in *E*. *granulosus* cysts

A UV spectrophotometric method was used for the estimation of intracystic Glb concentrations, which is based on measurement of absorption at a maximum wavelength of 242 nm [25]. Hydatid cyst fluid was extracted from metacestodes incubated under control conditions or treated with Glb (10, 50, 100 and 200 μM) for 5 days. Then, 500 μl of hydatid liquid from each sample were mixed with 2 ml chloroform and centrifuged at 7500 xg for 1 min, the supernatants were discarded, and the absorbance of the chloroform phase was measured at 242 nm. A standard curve was prepared using a double spectrophotometer (Shimatzu-UV-100) and different concentrations of pure Glb dissolved in hydatid liquid, which obeyed Beer’s law in the range of 5–30 μg/ml.

Since both Glb and atorvastatin (ATV) are transported by OATPs in humans, we also analyzed the ATV uptake in *E*. *granulosus* cysts. For that, cysts were incubated with ATV (10 and 50 μM) and the drug uptake was determined by the spectrophotometric method above described using an ATV (5–30 μg/ml) standard curve [26].

### Experimental animals and determination of efficacy of *in vivo* treatments

Healthy female CF-1 mice (30–35 g, 8 weeks old) supplied by the SENASA, Mar del Plata were acclimatized for one week before initiation of the experiment. Mice were infected by intraperitoneal infection with 1,000 protoscoleces in 0.5 ml of medium 199 to produce experimental secondary hydatid disease [23]. The animals were maintained in standard polyethylene cages (five mice per cage), under controlled laboratory conditions (temperature 20±2°C, 12 hour light/12 hour dark with lights off at 8.00 p.m., 50±5% humidity). Food and water were provided *ad libitum*. Every 3 days, animals were placed into a clean cage with fresh sawdust. All the pharmacological treatments were performed by intragastric administration of a drug suspension (0.3 ml/animal). At the end of experiments, mice were euthanized by cervical dislocation and previous anesthesia with ketamine–xylazine (50 mg/kg/mouse– 5 mg/kg/mouse). All efforts were made to minimize suffering. A minimum number of animals was used in each experiment. At necropsy, the peritoneal cavity was opened, the hydatid cysts were carefully recorded, and the weights were determined from each animal. The efficacy of treatments was calculated using the following formula: 100 x {(mean cyst weight of control group)–(mean cyst weight of treated group)}/ (mean cyst weight of control group). In addition, samples were processed for scanning electron microscopy (SEM) with a JEOL JSM-6460LV electron microscope as previously described [22].

### Therapeutic effectiveness of glibenclamide and its combination with albendazole

At 2 months post-infection (p.i.), mice were randomly assigned into four groups of 10 animals each. Drugs were administered by oral gavage daily for 60 days as follows: control group (receiving corn oil as a placebo), ABZ at 5 mg/kg/day, Glb at 5 mg/kg/day, and a combination of ABZ (5 mg/kg/day) plus Glb (5 mg/kg/day). At the end of the treatment period, animals were euthanized and necropsy was carried out immediately thereafter.

### Sequences analysis of *Echinococcus* organic anion-transporting polypeptide

Given that Glb is a substrate for OATPs, BLASTp searches for OATP homologs in the *E*. *granulosus* genome database (http://www.sanger.ac.uk/Projects/Echinococcus, [27]) were carried out using *Mus musculus* and *Homo sapiens* orthologs as queries. These data allowed the identification of two putative orthologous genes of OATP whose predicted open reading frame were analyzed. Orthologs were selected based on reciprocal best BLAST hits [28, 29] on an E-value cut-off of 1xe^-25^ and on the presence of the characteristic domains in the deduced amino acid sequences. Sequence alignments were generated with the CLUSTALX software program and modeling of secondary structure of the putative receptor was obtained from the deduced primary structure using Gen-THREADER (http://bioinf.cs.ucl.ac.uk/psipred/). The prediction of transmembrane regions was analyzed with TMHMM Server v. 2.0 (http://www.cbs.dtu.dk/services/TMHMM), SACS HMMTOP program (http://www.sacs.ucsf.edu/cgi-bin/hmmtop.py) and TOPO2 (http://www.sacs.ucsf.edu/cgi-bin/open-topo2.py).

### Statistics

Data within experiments were compared and significance was determined using the student’s t test and the non-parametric Mann-Whitney test. All data were shown as arithmetic mean ± S.D. and *p* values are indicated in each assay.

## Results

### Glibenclamide decreases viability of *E*. *granulosus* protoscoleces *in vitro*

**Fig 1:**
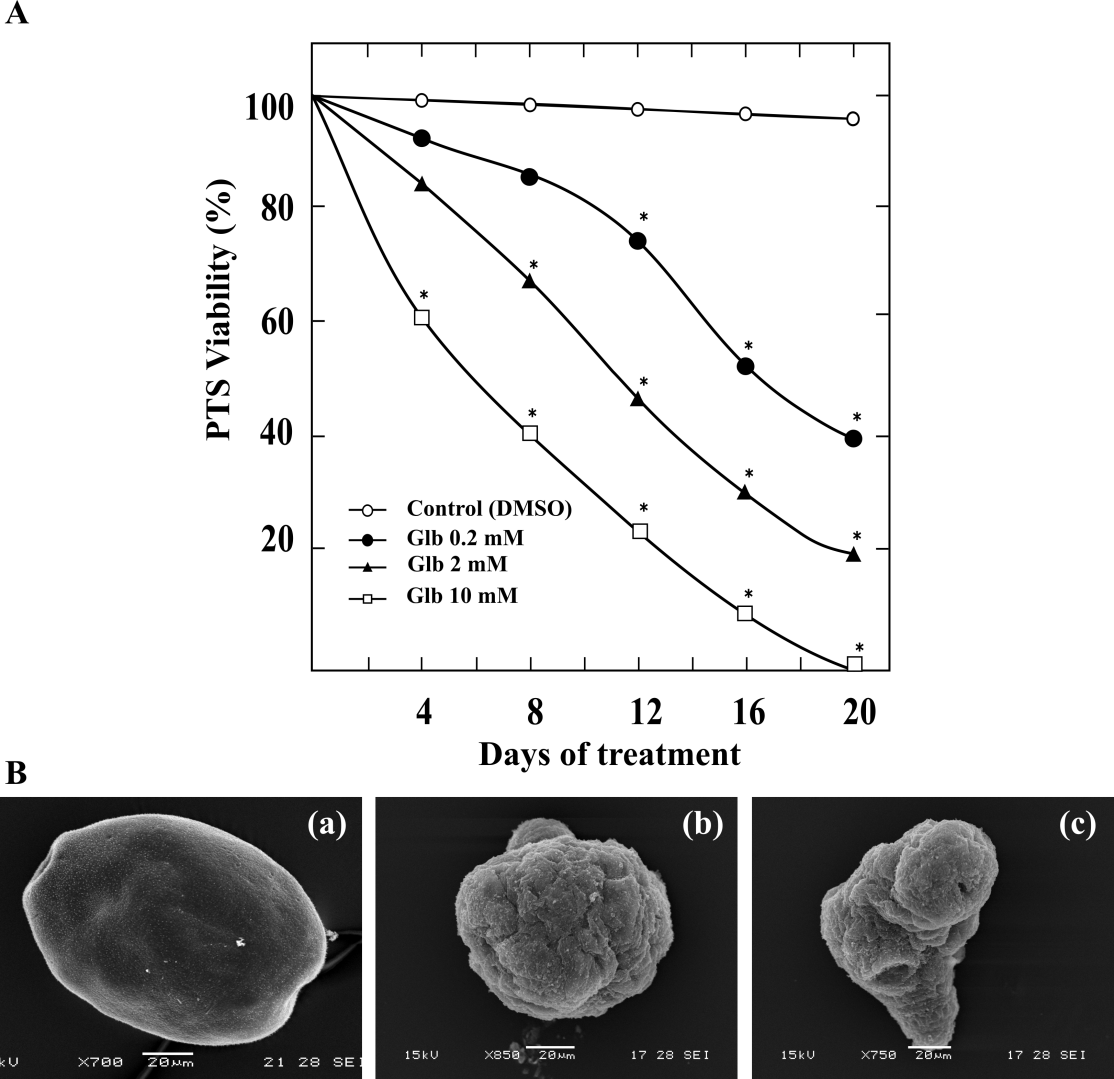
*In vitro* effect of glibenclamide on viability of *E*. *granulosus* protoscoleces. (A) Viability of protoscoleces incubated for 20 days with 0.2, 2 and 10 mM of glibenclamide (Glb). Parasites incubated in culture medium containing DMSO served as controls. The protoscolex viability was determined every two days by the methylene blue exclusion test (at least 100 protoscoleces per replica were counted each time). Data are the mean ± S.D. of three independent experiments. *Statistically significant difference (*p* < 0.05) compared with control. (B) Representative SEM images of control protoscoleces (a) or treated with 10 mM Glb for 7 days (b, c). (a) Invaginated control protoscolex; (b, c) Evaginated treated protoscoleces, with altered tegument in the soma region and loss of microtrichias on the escolex region. Bars indicate: 20 μm.

To investigate the *in vitro* effect of Glb on the viability of *E*. *granulosus* protoscoleces, the percentage of dead parasites was analyzed in response to various Glb concentrations. As shown in Fig 1A, Glb exposure led to a significant dose- and time-dependent decrease in the viability of protoscoleces. The mortality rate reached 100% during the treatment with 10 mM Glb at day 20, whereas parasites treated with 2 mM or 0.2 mM Glb showed a mortality rate of 80% and 60%, respectively. Control parasites remained at least 95 ± 5.0% viable during the complete experiments. In addition, SEM studies demonstrated the unaltered structure of control larvae and the drug-induced ultrastructural damage on treated parasites (Fig 1B). After 7 days of treatment with 10 mM Glb, the soma region was contracted (Fig 1Bb) and loss of hooks, shedding of microtriches and sucker deformation were observed (Fig 1Bb-c).

### Glibenclamide causes mitochondrial membrane depolarization in protoscoleces

Given that Glb could dissipate the mitochondrial membrane potential [21], we studied the mitochondrial functional status using the ΔΨm indicator JC-1 in Glb-treated protoscoleces. JC-1, a positively charged fluorescent compound, can penetrate mitochondria and change its color as a function of ΔΨm. It accumulates as aggregates with intense red fluorescence within the mitochondria when the ΔΨm is high, or remains as green monomers in the cytoplasm and the mitochondria when the ΔΨm is low [30].

**Fig 2:**
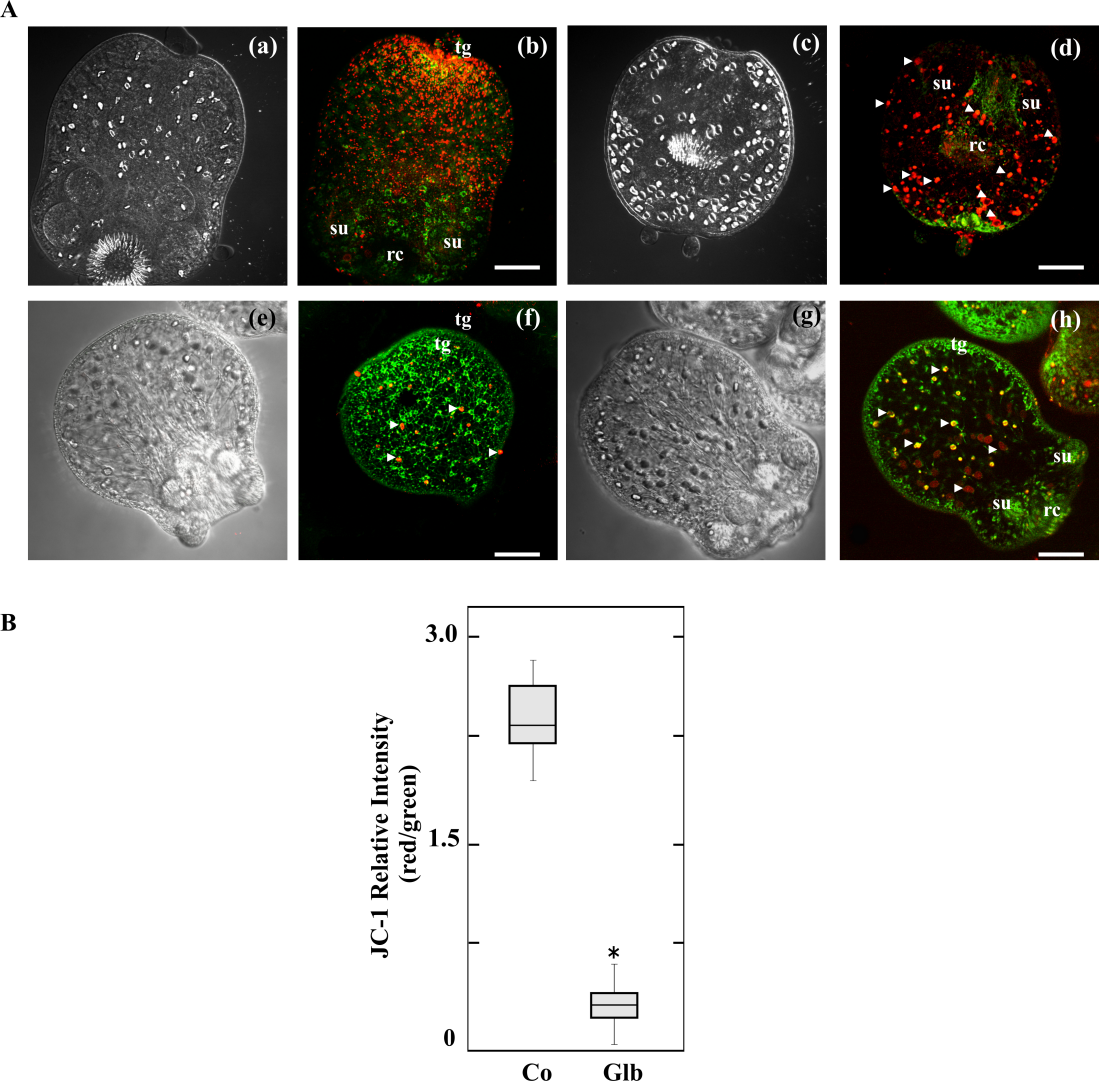
Mitochondrial membrane potential in glibenclamide treated-protoscoleces. (A) Representative confocal images showing JC-1 fluorescence in protoscoleces incubated under control conditions (a-d) or treated with 200 μM glibenclamide (Glb, e-h) for 24 h. tg: tegument; rc: rostellar cone; su: sucker. Bars indicate 50 μm. Red and yellow dots indicated by arrowheads correspond to stained calcareous corpuscles. (B) Boxplot graph showing the values of the red/green JC-1 fluorescence ratios measured in control (Co) and Glb-treated protoscoleces by Image J Software. *Statistically significant difference (*p* < 0.05) compared with control.

Control and Glb-treated protoscoleces were examined by confocal microscopy for JC-1 fluorescence. Following 24h treatment with 200 μM Glb, the relative values of red/green JC-1 fluorescence ratios showed low dispersion. At this point, untreated protoscoleces showed a ratio of red to green fluorescence with a mean value of 2.3 (Fig 2Aa-d and 2B), whereas Glb treated protoscoleces showed a lower mean ratio of around 0.3 (Fig 2Ae-h and 2B). Glibenclamide treatment induced an increase in depolarized regions indicated by the disappearance of red fluorescence and the increase of green fluorescence (Fig 2Af,h).

### Glibenclamide increases intracellular calcium levels in protoscoleces

**Fig 3:**
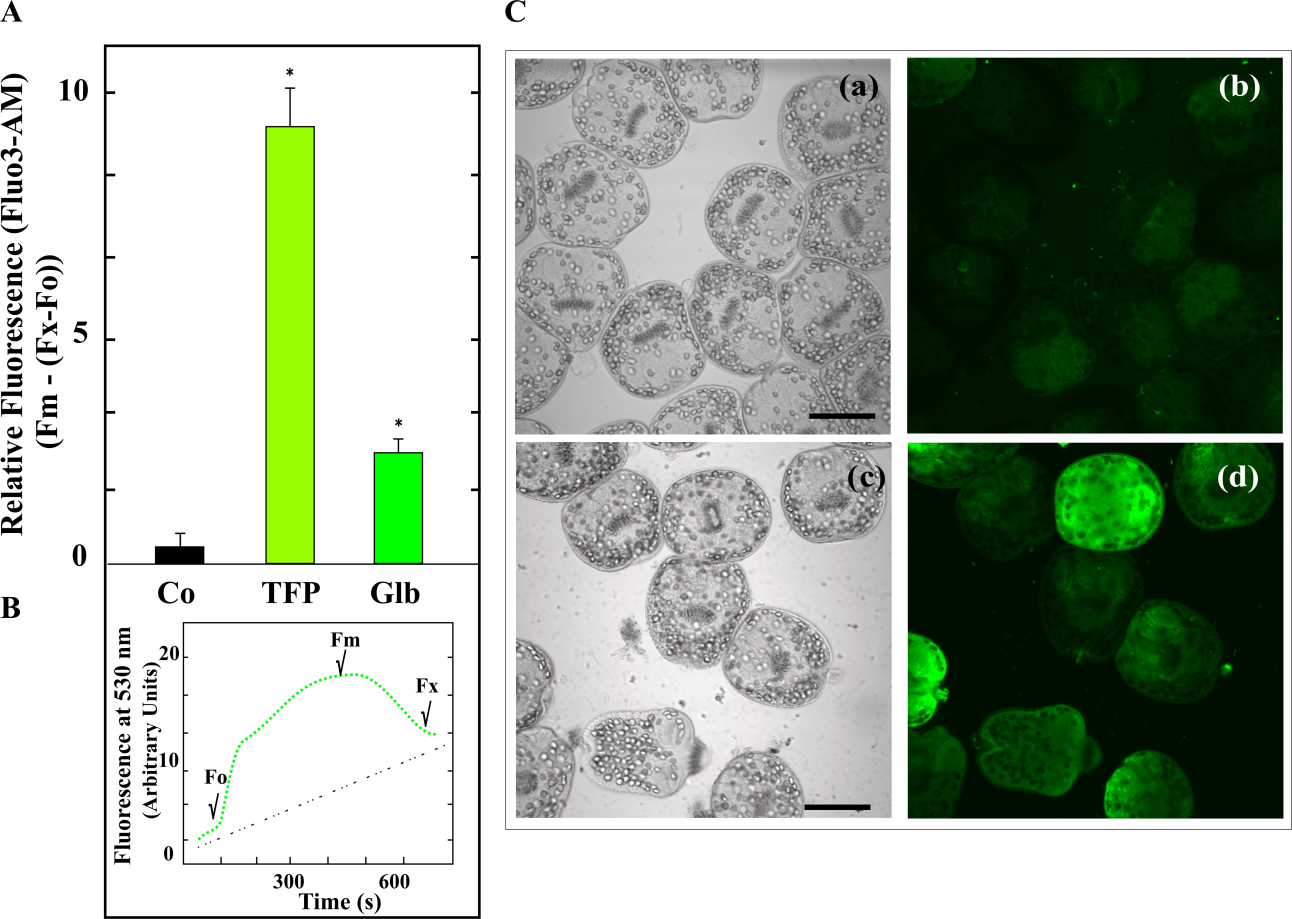
Effect of glibenclamide on intracellular calcium levels in *E*. *granulosus* protoscoleces. (A) Protoscoleces were incubated with buffer (Co), 200 μM glibenclamide (Glb) or 10μM trifluoperazine (TFP, positive control) for 2 h and then loaded with Fluo3-AM ester solution in darkness. Data are the mean ± S.D. of three independent experiments. *Statistically significant difference (*p* < 0.05) compared with control. (B) Free calcium profiles in protoscoleces. Prototype kinetics shown the fluorescence increase with Fluo3-AM. Fo, initial fluorescence; Fm, maximum fluorescence; Fx, final fluorescence. The graphic shows the free calcium decay to baseline within about 600 s with a partial recovery of the initial fluorescence which reveals a strong gradient in the calcium signal during the treatment of this puricellular organism with Glb. (C) Confocal imaging reveled intracellular calcium increase in Glb-treated protoscoleces. Photomicroscopy of light field (a, c) and of fluorescence field (b, d). Control (a, b); protoscoleces treated with Glb (c, d). Bars indicate: 100 μm.

Membrane depolarization induced by Glb would result in the opening of voltage-gated Ca^2+^ channels, inducing changes in the levels of intracellular Ca^2+^ [20, 31]. Based on this and on the depolarizing effect of Glb on mitochondria, [Ca^2+^]_i_ were determined in protoscoleces treated with 200 μM drug. Glibenclamide exposure showed an increase of three-fold in free [Ca^2+^]_i_ over a 2 h observation period, compared with the control (Fig 3).

### Glibenclamide and its combination with albendazole sulfoxide affect viability of *E*. *granulosus* metacestodes *in vitro*

**Fig 4:**
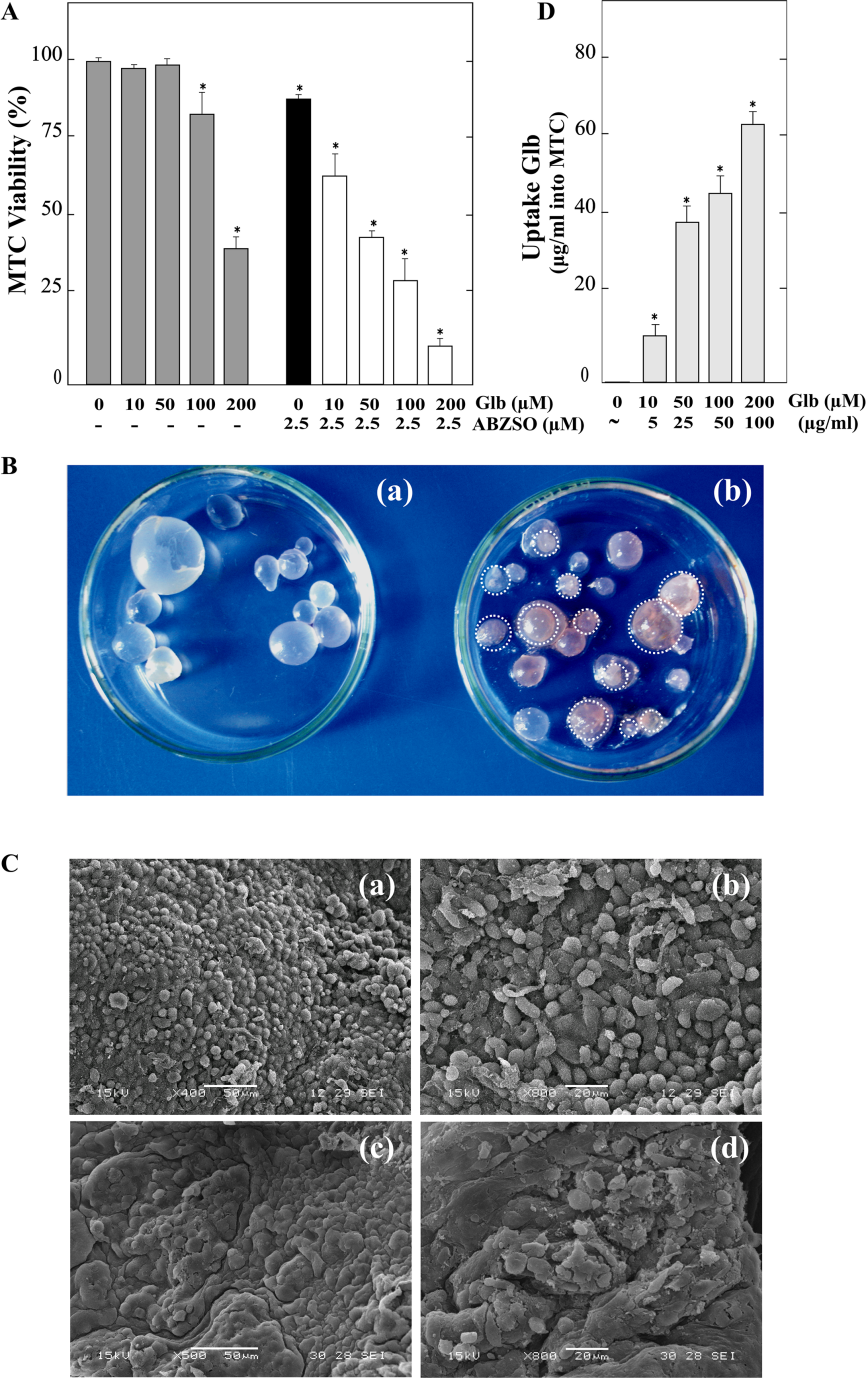
*In vitro* effect of glibenclamide and its combination with albendazole sulfoxide on viability of *E*. *granulosus* metacestodes. (A) Viability of metacestodes (MTC) incubated for 5 days with 10, 50, 100 and 200 μM of glibenclamide alone (Glb), 2.5 μM albendazole sulfoxide alone (ABZSO) and 10, 50, 100 and 200 μM Glb plus 2.5 μM ABZSO in combination. Parasites incubated in culture medium containing DMSO served as controls. Data are the mean ± S.D. of three independent experiments. *Statistically significant difference (*p* < 0.05) compared with the appropriate control (without drug or ABSZO alone). (B) Macroscopic damage in metacestodes exposed to glibenclamide. Control metacestodes (a) without morphological changes and metacestodes treated with 200 μM Glb for 5 days (b) showing increased permeability and collapsed germinal layer (circles). (C) Representative SEM images of control metacestodes (a, b) and treated with 200 μM glibenclamide for 5 days (c, d). (a, b) Control metacestode with an intact germinal layer; (c, d) Treated metacestode with altered germinal layer. Bars indicate: 50 μm in (a) and (c), and 20 μm in (b) and (d). (D) Intracystic concentrations of Glb from cysts incubated with 10, 50, 100 and 200 μM Glb in the experiment indicated in (A). *Statistically significant difference (*p* < 0.05) compared with control.

The anti-echinococcal activity of Glb was also tested in *E*. *granulosus* metacestodes maintained *in vitro* for 7 days. After incubation with 200 μM Glb, metacestodes presented detachment of the germinal layer in ~ 60% and 100% of the cysts at days 5 and 7, respectively (Fig 4A and 4B). Interestingly, the decrease in cyst viability with the combination of Glb and ABZSO was more pronounced, reaching 60% with 50 μM Glb + 2.5 μM ABZSO and 90% with 200 μM Glb + 2.5 μM ABZSO after 5 days of treatment (Fig 4A). Conversely, the viability of metacestodes incubated with 2.5 μM ABZSO was 85% at day 5, whereas control metacestodes remained at least 90% viable throughout the experiment. Studies by SEM revealed that control metacestodes exhibit no ultrastructural alterations in parasite tissue, showing an intact germinal layer comprised of a multitude of different, morphologically intact, cell types (Fig 4Ca,b). In contrast, Glb-treated metacestodes revealed loss of the typical multicellular structure (Fig 4Cc,d).

Furthermore, Glb concentration was measured in cysts incubated with 10, 50, 100 and 200 μM Glb using hydatid liquid from untreated cysts as negative control. Mean intracystic drug concentrations were 10 ± 3 μg/ml, 38 ± 5 μg/ml, 43 ± 6 μg/ml and 62± 4 μg/ml, respectively (Fig 4D). Thus, the drug was concentrated relative to the culture medium in the treatments in which there was no membrane detachment (10 and 50 μM Glb, equivalent to ~5 and ~25 μg/ml drug in the culture medium), but not in those in which there was membrane detachment (100 and 200 μM Glb, equivalent to ~50 and ~100 μg/ml drug in the culture medium). The intracystic drug accumulation may be suggesting the presence of Glb transporters in the parasite. In order to investigate this possibility, we analyzed the presence of OATPs in *E*. *granulosus*. Extensive BLASTp searches on the available parasite genome revealed two genes coding for members of the OATP family (GeneDB systematic names EgrG_000970200 and EgG_000345700). Due to the high identity of these predicted proteins (GenBank accession numbers CDS16984 and CDS22241), with vertebrate OATP orthologs, their genes were named Eg-*oatp*-1 and Eg-*oatp*-2. The genes encode a 1019 and a 1040-amino acid protein, respectively, both of which show a membrane topology in accordance with the prototype transporter, which includes 10±12 α-helical transmembrane domains (TMDs) and a large extracellular loop between TMDs IX and X [32, 33]. The Eg-OATP-1 and Eg-OATP-2 sequences aligned with ~ 27% and ~ 26% identity with the *Homo sapiens* ortholog (GenBank accession number AAH41095), respectively (S1 Fig). Moreover, the transcriptional expression of both genes was confirmed in different parasite stages [34]. However, although the transcript corresponding to Eg-*oatp*-1 was detected as a single sequence (GenBank accession number: EUB57978.1), the corresponding to Eg-*oatp*-2 was detected as four sequences (GenBank accession numbers: EUB58511, EUB55076.1, EUB55077.1 and EUB55078.1). It also is noteworthy that the transcript of Eg-*oatp*-1 was annotated as NEDD8-activating enzyme E1 catalytic subunit.

Additionally, we examined the possible role of Eg-OATPs in ATV transportation in metacestodes. For that, drug uptake was determined by the spectrophotometric method above described, since the maximum absorbance of ATV is also observed at ~242 nm [26]. Atorvastatin concentration was between 50 and 125 μg/ml in cysts treated with drug concentrations in the range of 10–50 μM (equivalent to 5.6 and 28 μg/ml).

### Glibenclamide is effective against established cystic echinococcosis in mice

**Fig 5:**
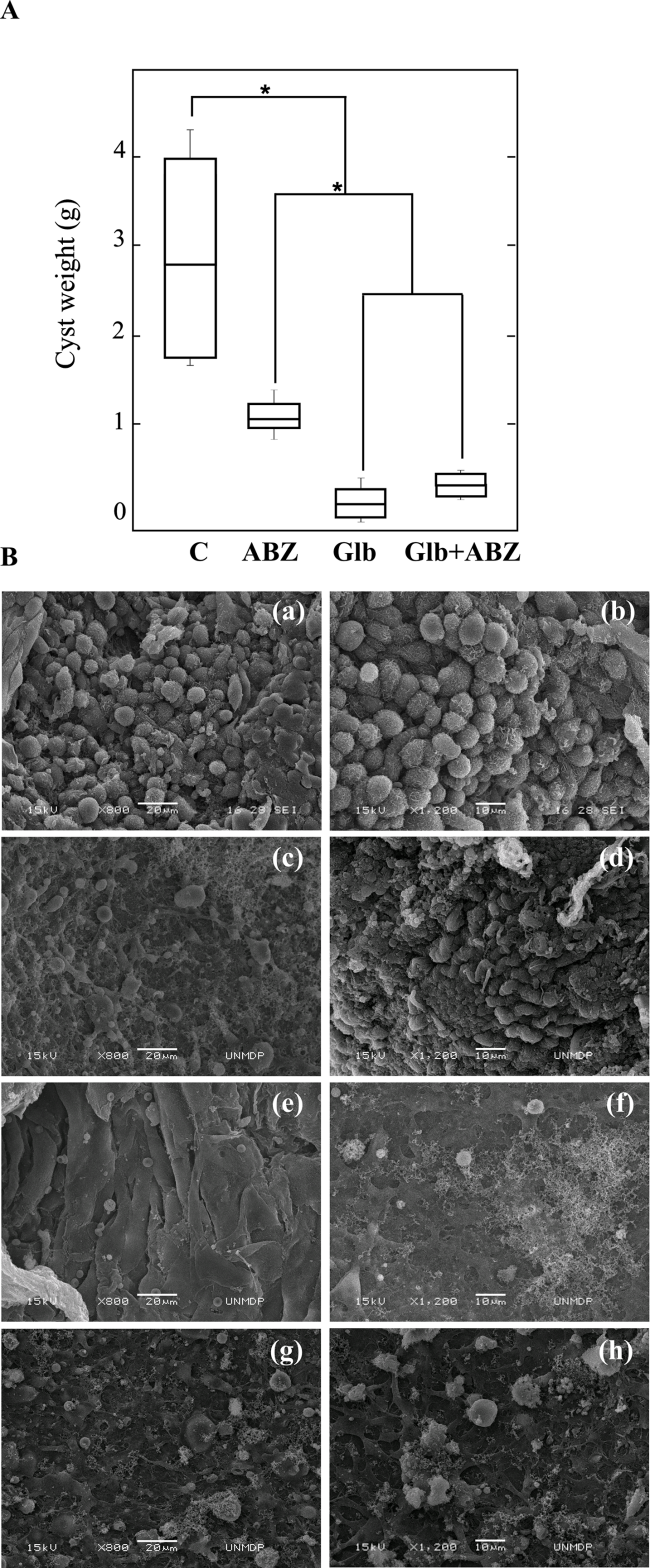
Therapeutic efficacy study in *E*. *granulosus* infected mice. (A) Box plots showing the comparative distribution of the weight (g) of cysts recovered from untreated mice (C) and treated with albendazole (ABZ, 5 mg/kg/d), glibenclamide (Glb, 5 mg/kg/d) and the combination of both drugs (Glb+ABZ) for 60 days. The weight of cysts was significantly decreased upon all treatments compared with the control (*p <0.05), but the decrease was more prominent in the groups receiving Glb alone or the combined treatment than that with ABZ alone (*p < 0.05). (B) Representative SEM images of hydatid cysts recovered from untreated mice (a, b) or treated with ABZ (c, d), Glb (e, f) and Glb+ABZ (g, h). Bars indicate: 20 μm in (a, c, e, g), and 10 μm in (b, d, f, h).

Based on our *in vitro* results, we then examined the *in vivo* therapeutic effect of Glb and ABZ on the growth of *E*. *granulosus* larval stage in the murine CE infection model. To do this, protoscoleces were intraperitoneally injected in CF1 mice and treated 2 months later by oral administration of vehicle, ABZ (5 mg/kg/day), Glb (5 mg/kg/day) or the combination of Glb plus ABZ (5 mg/kg/day plus 5 mg/kg/day) over a period of 60 days. All infected animals in this study developed hydatid cysts in their abdominal cavity. At 4 months p.i., every treatment from the therapeutic efficacy study (ABZ, Glb and ABZ plus Glb) resulted in a significant reduction (n = 10 *p* <0.05) of the cyst weights compared to those obtained from untreated mice (2.78 ± 0.310 g) (Fig 5A). Cysts developed in mice belonging to the combined therapy group (0.4 ± 0.01 g for Glb plus ABZ treatment) weighed significantly less (*p* < 0.05) than those from the group treated with ABZ alone (1.08 ± 0.040 g), but not than those from the group treated with Glb alone (0.2 ± 0.023 g)(Fig 5A). No adverse effects or weight change were observed in mice.

In order to analyze the ultrastructural changes of cysts recovered from the different treatments, SEM studies were performed. Cysts from control mice at 4 months p.i. appeared turgid, with a massive amount of intact cells in germinal layers (Fig 5Ba,b). In contrast, metacestodes collected from ABZ-, Glb-, or Glb+ABZ-treated mice displayed a marked reduction in the amount of germinal cells (Fig 5Bc-h), with the changes more pronounced in the groups receiving either Glb alone or the combined treatment as compared with the group receiving ABZ alone.

## Discussion

In an attempt to find new anti-echinococcosis drugs, the effectiveness of Glb against the larval stage of *E*. *granulosus* was evaluated because this drug has been shown to be active against other parasites. This drug prevented *in vitro* growth of *P*. *falciparum* by inhibiting the transport of low molecular weight solutes in infected human erythrocytes [35]. In addition, Glb decreased the viability of *Leishmania* sp. both *in vitro* and *in vivo*, and this effect seems to be related to its role on Ca^2+^ homeostasis [36–38]. *In vivo* effects of Glb would not depend on the reduction in glucose supply to the parasite, since under normoglycemic conditions, the chronic administration of the drug modified neither plasma insulin nor IGF-1 levels [6]. Additionally, the Glb acute administration (10 mg/day) did not alter endogenous glucose production [39]. In this report, we demonstrated that Glb kills *E*. *granulosus* protoscoleces and metacestodes in culture and reduces cystic weight in a murine secondary hydatidosis model. The drug toxicity mechanism could be related to the mitochondrial membrane depolarization and the increase of [Ca^2+^]_i_ detected in the parasite.

Glibenclamide reduced *in vitro* viability of protoscoleces and metacestodes in a dose- and time-dependent manner (Figs 1 and 4). It should be noted that the tegumental uptake system and tissue compartmentalization are decisive aspects affecting the access of anthelmintic molecules to target sites in helminth parasites [40]. Particularly for metacestodes, we used concentrations in the range reported in other *in vitro* studies with mammalian cells [41]. However, higher concentrations were required for a pharmacological effect of the drug on protoscoleces, as it has been previously reported [22, 23, 24, 42]. The metabolism and the complexity of the histological structure of the protoscolex (differentiated into different tissues) compared to that of the metacestode (delimited by a thin germinal layer of parasite cells) may account for such a difference in drug susceptibility between both larval forms [23, 43]. In comparison to BZMs, Glb has a rapid *in vitro* protoscolecide action. Regarding albendazole and ABZSO, they decrease protoscolex viability to 50% after 25 days of treatment [44, 45]. However, when using 200 μM of Glb such decrease was achieved even before 20 days of incubation (Fig 1A). Furthermore, the effect of sulfonylurea (both alone and combined with ABZO) on the turgidity and collapse of metacestodes was evident earlier than in the case of ABZ combined with other drugs whose targets are ion channels, such as PZQ or ivermectin [45, 46]. In these latter reports, the rate of collapsed cysts exceeded 70% only after 10 days of treatment, whereas with 200 μM Glb + 2.5μM ABZSO approximately 90% of the cysts collapsed after 5 days of incubation (Fig 4). Similarly to ABZ, PZQ and ivermectin, Glb induced a contraction of the posterior region of the soma and a loss of microtriches in the scolex region in protoscoleces (Fig 1B) [44–46]. Moreover, loss of the characteristic multicellular appearance of the germinal layer was observed in metacestodes incubated with the drug (Fig 4C), as has been reported for modulating compounds of Ca^+2^ [42, 47]. Additionally, the results of *in vitro* assays in the presence of low Glb concentrations (at which membrane detachment was not observed, Fig 4A) showed drug accumulation in the cysts, thus suggesting the presence of transporters involved in the uptake of Glb in the parasite (Fig 4D). Given that OATPs are involved in the uptake of the drug into different human tissues, they fit as candidate proteins [48]. In addition, the intracystic accumulation of ATV (this work)—a known substrate of OATPs—also suggests the presence of these transporters [49]. In this work, we identified two sequences that encode members of the OATP family of *E*. *granulosus* (Eg-*oatp*-1 and Eg-*oatp*-2), which conserve the characteristic topology of the prototype transporter (S1 Fig) and are expressed in different *E*. *granulosus* stages [34]. Since human OATPs mediate cellular uptake of a wide variety of endogenous amphipathic organic compounds (such as bile salts, steroid conjugates, certain oligopeptides and thyroid hormones) as well as certain drugs [50], it would be interesting to study the functional activity of the Eg-OATPs in the uptake of Glb and host molecules.

In our *in vivo* experiment, Glb showed anti-echinococcal activity with a clear reduction in cyst weight compared with the unmedicated control mice (Fig 5). In these experiments, ABZ was used in combination with Glb as a strategy to enhance its therapeutic action against CE. However, the results indicate that Glb inhibited the growth of the parasite in a similar way to the combined treatment, with both treatments significantly more effective than the monotherapy with ABZ (Fig 5A). Likewise, the ultrastructure of the cysts extracted from treated mice with Glb and Glb + ABZ showed the damage in germinal layer with a higher absence of cells compared to those obtained from mice treated only with ABZ (Fig 5B). Therefore, unlike *in vitro* trials with ABZSO (Fig 4A), Glb + ABZ combined treatment was not more effective than the Glb monotherapy. In contrast to ABZ oral administration, which presents erratic absorption in the gastrointestinal tract, Glb is rapidly and completely absorbed (> 95%), reaching peak plasma levels (140–430 ng/ml) between 2 and 4 h [39, 51, 52].

Therefore, further research should focus on attempting to modify the ABZ formulations to highlight the potential synergistic parasiticidal effect of the Glb + ABZ combination suggested by our *in vitro* experiments. On the other hand, since the dose of Glb selected for *in vivo* tests (5 mg/kg/day) was ten times lower than the highest human dose recommended (20 mg/day, after considering the pharmacokinetic differences between humans -t_1/2_ ~ 8 h- and mice -t_1/2_ ~ 1 h-), the dose of Glb could also be adjusted, considering that the selected dose is ~ 600 times less than the 50% lethal dose for mice (3250 mg/kg) [11, 38]. The amount of Glb dosed in our *in vivo* experiment would reach lower plasma levels than the drug concentrations used in the *in vitro* assays. The fact that Glb has a high level of plasma protein binding [53], could favor its solubility in plasma, resulting in a more powerful effect of the drug *in vivo* than *in vitro*. The high *in vivo* potency of Glb could also be explained by the pleiotropic effects of this drug previously described in rodent models of other human diseases [54].

Given that the binding of Glb to SURx produces the closure of K_ATP_ channels, reducing cellular potassium efflux and thus favouring membrane depolarization and the increase of [Ca^2+^]_i_ [55, 56], we analyzed the presence of the two subunits that constitute K_ATP_ channels in the *E*. *granulosus* genome. Although a Kir subunit could not be detected, it was determined that the parasite encodes an ABCC protein (EgrG_000592100 of 1998 amino acids) with 37–25% identity to the *H*. *sapiens* SUR regulatory subunit (Q09428 of 1581 amino acids). This protein is annotated as a multidrug resistance-associated protein (MRP), but the gene transcript is most similar to a canalicular multispecific organic anion transporter 2 (GenBank accession number: EUB57766) [27, 34]. Therefore, the inhibitor effect of this drug on *Echinococcus* larval stage viability cannot be explained through the presence of a typical SUR in the parasite. However, it has been previously described that Glb can induce changes in membrane potential and calcium homeostasis through mechanisms independent of SUR [20, 21]. In line with this evidence, our results demonstrated mitochondrial membrane depolarization and increase of [Ca^2+^]_i_ in Glb-treated protoscoleces (Figs 2 and 3). These data allow us to suggest that Glb could induce changes in the membrane excitability and consequently mitotoxicity in this cestode [16, 17]. Further studies should be carried out in order to characterize the action mechanism of Glb in *Echinococcus* sp.

Since helminth infections are endemic in developing countries, we have explored the possibility of repositioning antidiabetic drugs such as metformin [23,57] and Glb (this work), given that these drugs attack energy-generating systems [19] interfering with the mitochondrial activity and the ATP generation of this parasite [58], with high safety for the normoglucemic host. Further studies are needed in order to thoroughly investigate the mechanism involved in the therapeutic response of the *E*. *granulosus* larval stage to treatment with Glb.

## Supporting information

S1 Fig Sequence analysis of *Echinococcus granulosus* organic anion-transporting polypeptides (Eg-OATPs). Multiple sequence alignment of OATP proteins. Consensus is indicated in the last line, total (uppercase letter), partial (lowercase letter), conservative changes (numeral), absence of consensus (dots) and gaps introduced to maximize the alignment (dashes). Eg-OATP-1 conserves the ten cysteine residues in the large extracellular loop between transmembrane domains IX and X (C^731^, C^735^, C^737^, C^746^, C^758^, C^762^, C^780^, C^782^, C^828^, C^832^ -grey boxes-), while Eg-OATP-2 conserves eight of the ten residues (C^767^, C^771^, C^786^, C^796^, C^843^, C^845^, C^873^, C^877^ -grey boxes-). Eg-OATP-2 also conserves one of the two extracellular consensus sites for *N*-linked glycosylation (N^334^ -asterisk-) in extracellular loop III-IV [48]. On the other hand, both Eg-OATP-1 and Eg-OATP-2 conserve an arginine residue involved in the uptake of different substrates (R^881^ and R^926^, respectively -arrowhead) [59]. In addition, Eg-OATP-2 conserves a histidine residue located at the extracellular side of the transmembrane domain III (His^289^-arrow-) [48]. GenBank accession numbers for the OATP orthologous protein are: Hs, *Homo sapiens* (NP_009187); Ms, *Mus musculus* (NP_001239459); Eg, *Echinococcus granulosus* (CDS16984 and CDS22241).
(TIF)
