# Supplementary figures and images for: Anthelminthic activity of glibenclamide on secondary cystic echinococcosis in mice
Source: PLoS Negl Trop Dis. 2017 Nov 30;11(11):e0006111. doi: 10.1371/journal.pntd.0006111 (PMC5726723; doi:10.1371/journal.pntd.0006111)

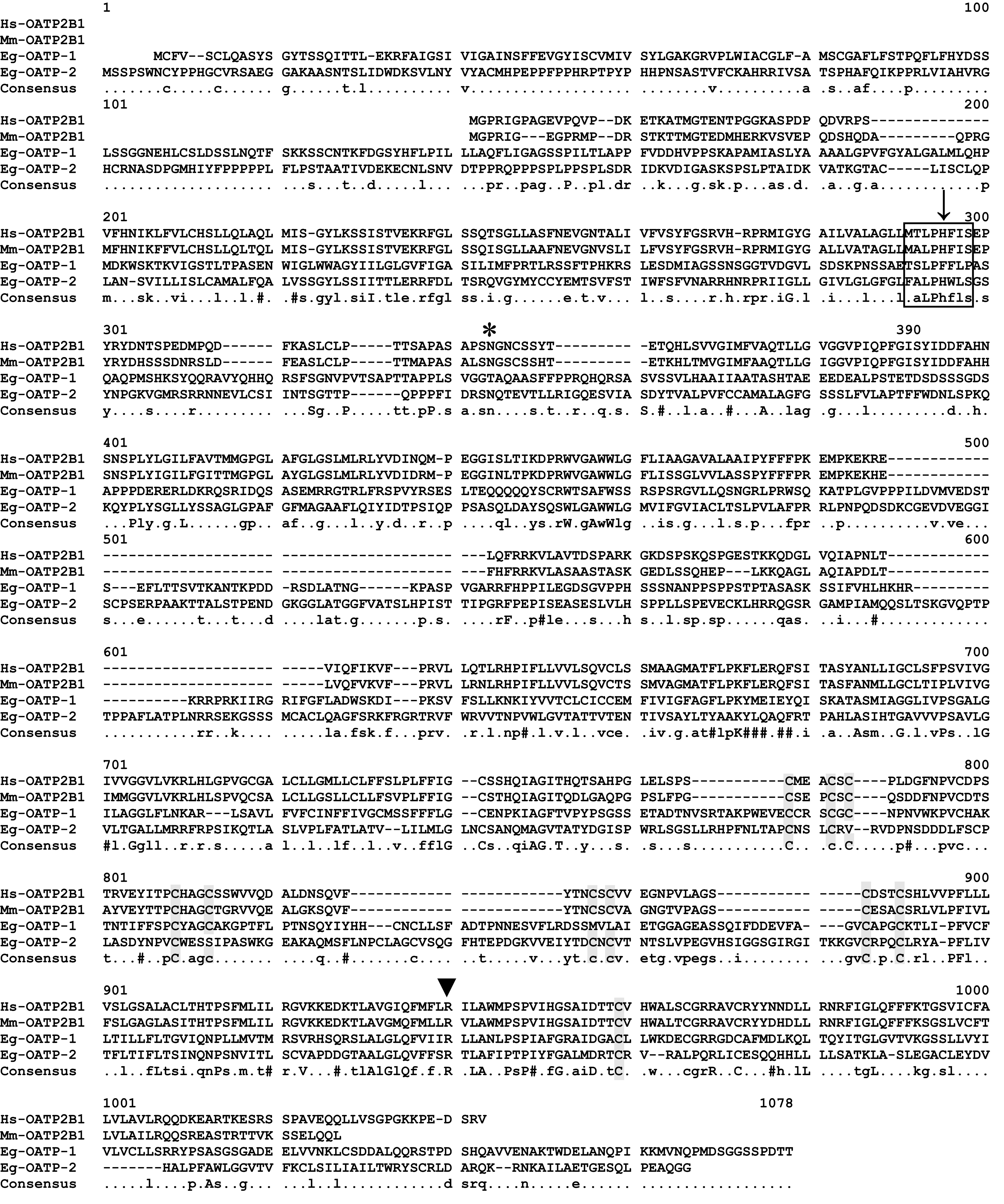

Supplement: S1 Fig — Multiple sequence alignment of OATP proteins. Consensus is indicated in the last line, total (uppercase letter), partial (lowercase letter), conservative changes (numeral), absence of consensus (dots) and gaps introduced to maximize the alignment (dashes). Eg-OATP-1 conserves the ten cysteine residues in the large extracellular loop between transmembrane domains IX and X (C731, C735, C737, C746, C758, C762, C780, C782, C828, C832 -grey boxes-), while Eg-OATP-2 conserves eight of the ten residues (C767, C771, C786, C796, C843, C845, C873, C877 -grey boxes-). Eg-OATP-2 also conserves one of the two extracellular consensus sites for N-linked glycosylation (N334 -asterisk-) in extracellular loop III-IV [48]. On the other hand, both Eg-OATP-1 and Eg-OATP-2 conserve an arginine residue involved in the uptake of different substrates (R881 and R926, respectively -arrowhead) [59]. In addition, Eg-OATP-2 conserves a histidine residue located at the extracellular side of the transmembrane domain III (His289-arrow-) [48]. GenBank accession numbers for the OATP orthologous protein are: Hs, Homo sapiens (NP_009187); Ms, Mus musculus (NP_001239459); Eg, Echinococcus granulosus (CDS16984 and CDS22241). (TIF) [file pntd.0006111.s001.tif]
